# Contractile dynamics change before morphological cues during florescence illumination

**DOI:** 10.1038/srep18513

**Published:** 2015-12-22

**Authors:** S. G. Knoll, W. W. Ahmed, T. A. Saif

**Affiliations:** 1 Department of Mechanical Science and Engineering, University of Illinois at Urbana-Champaign, Urbana, IL, USA; 2 Laboratoire Physico Chimie Curie, Institut Curie, PSL Research University, CNRS UMR168, 75005, Paris, France; 3 Sorbonne Universités, UPMC Univ Paris 06, 75005, Paris, France

## Abstract

Illumination can have adverse effects on live cells. However, many experiments, e.g. traction force microscopy, rely on fluorescence microscopy. Current methods to assess undesired photo-induced cell changes rely on qualitative observation of changes in cell morphology. Here we utilize a quantitative technique to identify the effect of light on cell contractility prior to morphological changes. Fibroblasts were cultured on soft elastic hydrogels embedded with fluorescent beads. The adherent cells generated contractile forces that deform the substrate. Beads were used as fiducial markers to quantify the substrate deformation over time, which serves as a measure of cell force dynamics. We find that cells exposed to moderate fluorescence illumination *(λ* = 540–585 nm, *I* = 12.5 W/m^2^, duration = 60 s) exhibit rapid force relaxation. Strikingly, cells exhibit force relaxation after only 2 s of exposure, suggesting that photo-induced relaxation occurs nearly immediately. Evidence of photo-induced morphological changes were not observed for 15–30 min after illumination. Force relaxation and morphological changes were found to depend on wavelength and intensity of excitation light. This study demonstrates that changes in cell contractility reveal evidence of a photo-induced cell response long before any morphological cues.

Various imaging modalities are utilized to precisely visualize small-scale biological phenomena. Many of these techniques incorporate illumination with fluorescence excitation light. Photo-induced effects of illumination are well known to be a risk in live-cell fluorescence imaging. A recent report emphasized the importance of circumventing phototoxicity in imaging by balancing acquisition speed, resolution, and photodamage^1^.

All cells are intrinsically photosensitive^2^,^3^. However, cells vary widely in their ability to sustain normal function under light exposure, making experiments difficult to control. Morphology is a common indicator of cell viability. Evaluating morphological changes during light exposure to assess possible cytotoxicity is a common practice used widely across various experimental studies^4^,^5^,^6^,^7^,^8^,^9^,^10^,^11^,^12^,^13^,^14^. When affected by light, cells often exhibit morphologies such as: necrosis, blebbing, formation of multiple nuclei or vacuoles, and swelling of mitochondria^3^,^9^. However, in general, there is significant variability amongst cells, even within the same culture of a single cell line^5^,^15^, which complicates viability assessments. The ability to detect effects of illumination on cells also depends on the observation time. Many cells may not exhibit an adverse response until reaching a specific phase of the cell cycle^14^,^16^. Hence, fluorescence illumination often will not yield morphological changes during shorter time periods, on the order of minutes, even though adverse effects may be present. Thus, it is advantageous to continue observation for an extended time period following illumination in order to determine potential implications on cell viability.

Adverse cell changes due to illumination are commonly assessed in terms of morphology. However, adverse effects may occur before morphological changes become observable. To address this, we developed a quantitative technique to rapidly detect cell sensitivity to fluorescent excitation light during short illumination durations (≤1 minute), before morphological changes occur. Recent studies have described microscopy techniques that mitigate effects of excitation light on cells, such as multi-photon, spinning-disk confocal, light sheet, and controlled light exposure microscopy^8^,^17^,^18^,^19^,^20^,^21^. These techniques are important for minimizing photo-induced changes in cells. In this study, we quantify cell response to excitation light in widefield fluorescence microscopy, a commonly used experimental platform. Here, we employ photo-induced change in cell contractility to identify their response to fluorescent illumination. Cell response to excitation light is assessed by measuring the effect of short duration fluorescence exposure on fibroblasts cultured on elastic polyacrylamide (PA) gels embedded with fluorescent beads. Adherent cells continuously pull on their underlying substrate^22^,^23^ and bead motion allows quantification of substrate deformation, which serves as a measure of cell force dynamics^24^,^25^. Here, we have induced cell force relaxation by illuminating cells with fluorescent excitation light. As a result, we observe a transition from the natural contractile state of the cell to relaxation, which we use to characterize cell sensitivity to light. This technique is applicable to any study that utilizes fiducial markers in substrates to track cell behavior, such as PDMS^26^, microposts and pillars^27^, and three-dimensional culture systems^28^,^29^. While photo-induced cell changes depend on several parameters, this study identifies the onset of photo-induced cell sensitivity as the transition from cell contraction to relaxation during illumination. We observe this transition prior to distinct morphological changes due to exposure.

## Materials and Methods

### Hydrogel fabrication and cell culture

Polyacrylamide (PA) gels of varying elastic moduli (2, 5, 10 kPa) were utilized. Gel stiffness was modulated by mixing acrylamide and bisacrylamide according to specifications reported in a well-established protocol^30^. Cell force contractility studies commonly employ beads with excitation (ex) wavelengths in the range of 505 to 633 nm and emission (em) wavelengths between 515 and 720 nm^23^,^31^,^32^,^33^,^34^,^35^,^36^,^37^,^38^,^39^,^40^,^41^,^42^,^43^,^44^,^45^. We embedded red (580/605 nm ex/em) fluorescent beads (Fluospheres®, Life Technologies, Carlsbad, CA) of diameter 100 nm very near (within 1.5 μm of) the PA gel surface^46^ for high-resolution measurements of substrate deformation. *N*-hydroxysuccinimide (NHS) and 1-Ethyl-3-[3-dimethylaminopropyl]carbodiimide hydrochloride (EDC) (Thermo Scientific, Waltham, MA) were used to couple the extracellular matrix protein, fibronectin (Life Technologies, Carlsbad, CA), to the gel surface^47^. Cells were plated on PA gels and immersed in media comprised of Dulbecco’s Modified Eagle’s Medium (Corning, Corning, NY) (4.5 g/L glucose, 4 mM L-glutamine) supplemented with 10% fetal bovine serum (Sigma-Aldrich, St. Louis, MO) and 1% Penicillin Streptomycin (Corning, Corning, NY).

### Imaging

All experiments were performed using an Olympus IX81 microscope and 40× UApo N340 water immersion objective (NA 1.15) (Olympus America Inc., Center Valley, PA) mounted on a vibration isolation table (Newport Corporation, Irvine, CA). An environmental chamber enclosed the experimental platform, and maintained cell culture conditions throughout imaging (5% CO_2_, 70% humidity and 37° C). Images were acquired with a Neo sCMOS camera (active pixels 1392 × 1040, resolution of 165 nm per pixel) (Andor Technology, Belfast, Northern Ireland). A widefield fluorescent metal halide lamp (X-Cite® 120, Excelitas Technologies, Waltham, MA) coupled with an mCherry filter (Semrock Brightline mCherry-M-OMF, 540–585/600–682 nm ex/em, Rochester, NY) was utilized for fluorescence imaging. Samples were illuminated with fluorescent excitation light of intensity 12.5 W/m^2^ (as measured by a PM100 power meter (ThorLabs) at a sample plane 15.5 mm above the objective). Additional experiments were performed with either a neutral density filter (32ND25, Olympus America Inc., Center Valley, PA) coupled with the aforementioned widefield fluorescent metal halide lamp and mCherry filter (*λ* = 540–585/600–682 nm ex/em, *I* = 3.0 W/m^2^) or (2) a deep red collimated LED (*λ* = 640-680/650 (high pass) nm ex/em, *I* = 1.9 W/m^2^) (Thorlabs, Inc., Newton, NJ) to test the effect of higher wavelength and lower intensity on photo-induced cell force relaxation. For the remainder of this paper, the three excitation light sources will be referred to as follows: mCherry = widefield halide fluorescence + mCherry filter; mCherry + ND25 = widefield halide fluorescence + mCherry filter + ND25 neutral density filter; LED = deep red collimated LED.

### Experimental procedure

**Figure 1:**
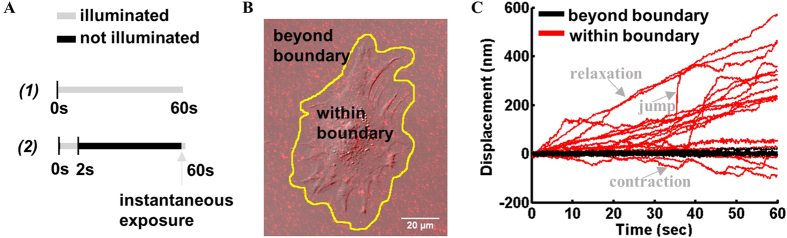
Overview of experimental methods. (**A**) Two illumination protocols for imaging fibroblast cells with mCherry excitation light: (1) continuous illumination for 60 s, and (2) continuous illumination for 2 s, followed by instantaneous illumination 58 s later for acquisition of a single frame at *τ* = 60 s. Illumination periods shown in grey. (**B**) Sample cell-beads overlay. Yellow outline indicates cell perimeter plus~5 μm. (**C**) Corresponding trajectories for a select number of beads within and outside of cell boundary. Example relaxation, contraction, and jump trajectories labeled in grey.

Monkey kidney fibroblast cells (ATCC, Manassas, VA) were plated sparsely (2,500 cells/cm^2^) on PA hydrogels and allowed to adhere and spread for 4 to 6 hours. An image of a single cell was acquired using differential interference contrast (DIC). Cells were subsequently illuminated with fluorescent excitation light while maintaining the same field of view. We utilized two illumination protocols to test the time sensitivity of cell response to excitation light: (1) continuous illumination for 60 s and (2) continuous illumination for 2 s followed by a single exposure at *t* = 60 s. A video of the substrate-embedded beads was recorded at a sampling rate of 10 Hz. The two illumination protocols are shown in Fig. 1A. This imaging procedure was repeated for gels of varying stiffness (n = 17 cells per stiffness). We quantified cell-induced substrate deformation with ±10 nm spatial resolution (Fig. S1) by measuring the displacements of beads within the gel using a single-particle tracking algorithm^48^. Trajectories of embedded beads were separated into two categories based on their position within the spread area boundary of a cell or outside (Fig. 1B). Bead motion away from the cell centroid is positive (relaxation), and bead motion towards the cell centroid is negative (contraction). In order to compare photo-induced changes in force contractility and morphology, cells were imaged with DIC immediately before and after illumination by mCherry excitation light. Change in morphology was assessed by the following visual cues: decrease in spread area, blebbing, and retraction of the cell edge. Only one cell per PA hydrogel was utilized to avoid repeated illumination of surrounding cells.

## Results and Discussion

### Substrate deformations indicate force relaxation

Single particle tracking of the embedded beads revealed nanoscale cell-induced deformations of the substrate on the order of tens to hundreds of nanometers (Fig. 1C). Most displacements followed a linear trajectory, representing a constant rate of relaxation (outward bead motion) or contraction (inward bead motion) of the cell. Occasionally, a continuous push or pull on the substrate was interrupted by an abrupt outward jump in displacement.

**Figure 2:**
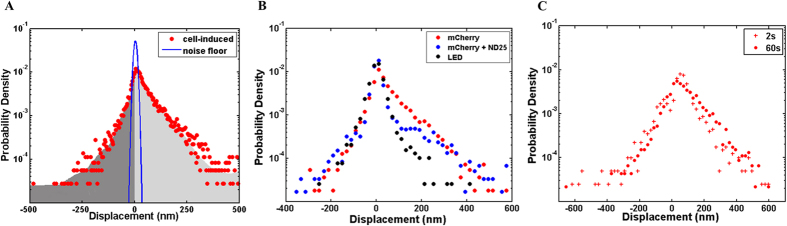
Probability distributions of cell-induced displacements for various experimental conditions. (**A**) Cell-induced displacements indicate cell force greater than noise floor. Probability distributions (*t* = 60 s) of bead displacements during one-minute illumination. Displacements induced by cells (n = 17) plated on 2 kPa substrate shown in red (*σ* = 41.4 nm). Gaussian central region represents noise floor of stationary beads in a gel with no cells, shown in blue (*σ* = 7.5 nm). The shaded regions under the distribution denote relaxation (light grey) and contraction (dark grey) of the substrate due to changes in cell force during illumination. (**B**) Cell force relaxation during illumination decreases with decreasing intensity. Probability distribution functions (*τ* = 60 s) for bead displacements as a result of cell force during illumination by various light sources (mCherry = widefield halide fluorescence + mCherry filter; mCherry + ND25 = widefield halide fluorescence + mCherry filter + ND25 neutral density filter; LED = deep red collimated LED). Each curve represents cell-induced motion of >2500 beads from n = 6 cells. The variance of the mCherry and both the mCherry + ND25 and LED distributions were determined to be statistically different according to an F-test (α = 0.05). (**C**) Effects of short and long (2 and 60 s) duration excitation light on substrate deformation due to cell forces at the 60th second. Probability distribution of displacements (*τ* = 60 s) for cells subject both protocols (Fig. 1A) with mCherry excitation light as denoted by figure legend, on a 2 kPa gel substrate. Each distribution is a representative data set for six cells. The variance of both distributions were determined to be statistically similar according to an F-test (α = 0.05).

To illustrate substrate deformation, a probability distribution function (PDF) was employed to quantify cell-induced displacement fluctuations (Fig. 2A). Gel substrates without cells were recorded to establish the experimental noise floor (blue curve in Fig. 2A). Deviation of the cell-induced displacements from the noise floor represents activity-driven motion generated by cell forces. The cell-induced displacement distribution is shifted right, indicating an overall outward motion representing cell relaxation (Fig. 2A). Skewness of the distribution 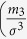, where 
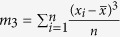
 and *σ* = St. Dev.) was quantified as 1.29 by computing the third moment about the mean. A skewness value greater than 1 indicates a skewed probability distribution^49^. Thus, motion is highly skewed toward positive, outward displacements (represented by the light gray area in Fig. 2A). By integrating the PDF, we find that the probabilities of outward and inward motion are 0.75 and 0.25, respectively. Similar results were observed on 5 and 10 kPa substrates (Table S1). Collectively, these data show the majority presence of outward motion during illumination indicating cell relaxation. For comparison, we performed the same experiments using lower intensity light (Fig. 2B). Decreased illumination intensity leads to decreased cell relaxation (Fig. 2B). This shows that the degree of cell force relaxation during illumination depends on exposure intensity. As such, the probability of relaxation, (*P*(*r*)), decreases with decreasing light intensity (Table S2). The high (>0.5) *P(r)* relative to probability of contraction (*P*(*c*) = 1 − *P(r)*) upon illumination for all tested excitation light sources suggests cell force relaxation occurs during light exposure. However, this effect is most prominent for higher intensity light. Cells illuminated with the mCherry excitation light both with and without the ND25 filter exhibit a strong bias toward relaxation, while those illuminated with the LED produce more symmetric displacement distributions indicating fluctuation around a mean contractile state (Table S2). This suggests that the LED light source causes the least cell perturbation.

Two illumination protocols were employed in order to investigate the time to onset of photo-induced cell relaxation: (1) continuous illumination for 60 s and (2) continuous illumination for 2 s, followed by a single exposure at *t* = 60 s (Fig. 1A). The purpose of protocol (2) was to evaluate the effect of short duration exposure to light. The net magnitude of displacements ((Δx^2^ + Δy^2^)^1/2^) was computed for cells subject to the two illumination protocols, and determined to be statistically similar (Fig. 2C). Further exposure to light during 60 s appears to have minimal additional effects on the initiated processes. Thus, photo-response is irreversibly activated within 2 s of exposure.

**Figure 3:**
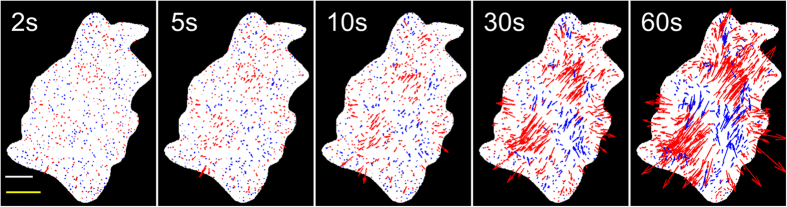
Cell forces relax along a distinct direction during illumination period. Blue and red arrows indicate inward and outward motion relative to the area centroid, respectively. Within 10 s of illumination, bead displacements become aligned. Increasing magnitude and presence of red arrows between 10–30 s shows increasing outward motion during illumination, representing force relaxation. Arrows representing displacement magnitude are magnified 50× to aid visual clarity. Width of grey (corresponding to the cell mask) and yellow (corresponding to the displacement vectors) scale bars represent 10 μm and 0.25 μm, respectively.

To examine the cell relaxation in more detail, we look directly at the displacement maps of embedded beads. Global displacement fields for all studied cells reveal the progression of force relaxation throughout illumination. Representative displacement fields for a cell exposed to 60 s of illumination are shown in Fig. 3. Arrows representing bead displacements during illumination became more aligned over time. This pattern was observed in all cells, and alignment of displacements was commonly oriented along the long axis of the cell. Alignment of bead displacements was observed for cells on various stiffness substrates (Fig. S3), although the alignment occurred later for stiffer substrates. Relaxation was assessed by a majority of motion outward relative to inward. This bias consistently increased throughout illumination (Fig. S4). The consistent relaxation during exposure indicates the presence of photo-induced cell contractility changes when illuminated with mCherry excitation light.

### Analysis of photo-induced change in dynamic cell contractility

To characterize the dynamics of photo-induced relaxation, cell-substrate deformations were modeled as an anisotropic contractile force dipole. Cell force relaxation primarily occurred along the long axis of the cell, thus the observed displacements (occurring at many spatial locations underlying a cell) were projected to a minimal configuration represented by two point-force locations. In the previously established force dipole theory, these two point-force locations physiologically correlate to adhesion sites^50^. Here, the two point-force locations correlate to the long axis of the cell, along which relaxation occurred.

**Figure 4:**
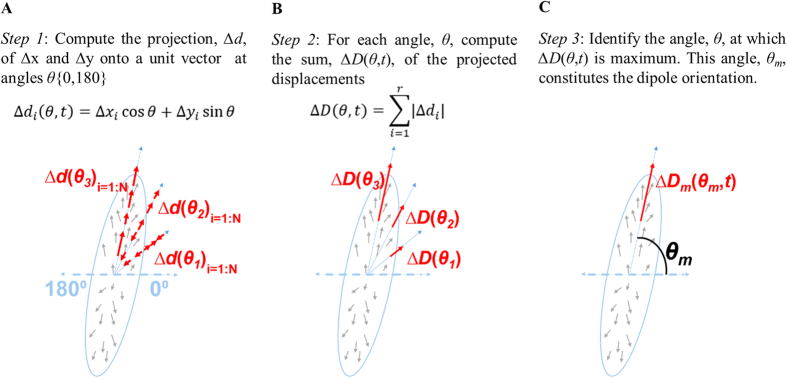
Dipole orientation identification process. Schematic representation of cell outlined in blue at a given time, *t*, during illumination. Localized displacements (at point locations 1 to *r*) shown by grey arrows. (**A**) Projections of all displacements onto a unit vector at angles *θ* {0,180}, given by Δ*d*. Three sample angles are shown. (**B**) All projections are summed to a single value, the dipole strength, Δ*D*. (**C**) Finally, the dipole orientation, *θ_m_*, is determined by the angle at which the maximum Δ*D* occurs.

The force dipole model is employed to provide a metric for cell force relaxation by assessing the global magnitude and orientation of substrate displacements over time. In order to quantify the spatiotemporal evolution and alignment of the deformation field during illumination, we define a net displacement dipole for the cell as a function of time. To define the displacement dipole, let (*x*_*i*_*(t)*, *y*_*i*_*(t)*) be the coordinates of bead *i* at time *t*. Although the beads are constrained in the substrate, they represent individual points that are displaced when the cell applies force. Let the displacements of each observed location *i* over time *t* be defined by


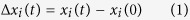


and


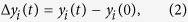


where exposure begins at *t* = 0. Let *n* be the unit vector oriented along *θ*{0, 180} with respect to the x-axis. The projections of Δ*x* and Δ*y* along *n* are then


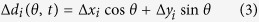


as shown in Fig. 4A. Then, the projected sum of the absolute displacements for all measured points under a cell is


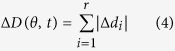


where *r* is the total number of point locations within the cell perimeter (Fig. 4B). The angle, *θ*_*m*_*(t)*, with unit vector, *n*_*m*_, that maximizes Δ*D(θ, t)*, is the direction of the displacement dipole at time *t* (Fig. 4C). The corresponding magnitude of the dipole, or the dipole strength, at time *t* is described by Δ*D*_*m*_*(θ*_*m*_*, t)*. A well-aligned displacement field will result in a Δ*D(θ, t)* that has a well-defined maxima when plotted as a function of *θ*. A randomly arranged displacement field will be represented by a Δ*D(θ, t)* with a broad distribution and no well-defined maxima. There may be multiple peaks of Δ*D(θ, t)*, representing multiple dipoles along *θ*_*j*_*(t), j* *=* 1, …., *K*, where *K* is the number of dipoles.

The sign of the dipole describes the state of cell force as either contraction (negative) or relaxation (positive). The time evolution of the dipole illustrates the evidence of cell relaxation observed during fluorescence illumination. The dipole sign is defined by the relative location of the two points that comprise the dipole, as projected onto *n*_m_. Each of the two points is characterized by either outward or inward motion. To determine the sign of the dipole, we first define the location, *d*_*im*_*(t)*, of a point, *i*, along *n*_m_ at time *t* as


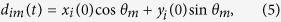


imposing the assumption that the initial point-force location remains constant relative to *t* = 0. Displacement, Δ*d*_*im*_*(t)*, of the same point, *i*, along *n*_m_ is


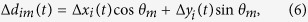


where a positive Δ*d*_*im*_*(t)*, implies that the point *i* has moved in the direction of *n*_m_ (outward) during time *t*. Let *M*_+_ and *M*_*-*_ be sum of the (total quantity of) outward and inward moving points at time, *t*, respectively, such that


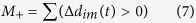



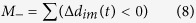


and *M*_+_ + *M*_−_ = *r*. Physically, a cell with a larger *M*_+_ than *M*_*−*_ value would characterize the state of that cell as force relaxation rather than contraction. The average spatial locations (centroid) of the two respective points whose outward and inward displacements define the dipole along *n*_m_ are given by *X*_*m*+_*(t)* and *X*_*m−*_*(t)*, respectively, where


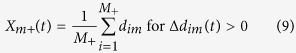


and


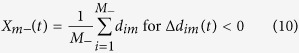


(Figs S5A–C). The net positive and negative displacements occurring at *X*_*m*+_ and *X*_*m-*_, respectively, are given by


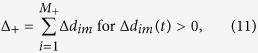


and


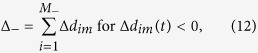


Then the displacement dipole, *D*_*p*_*(t)*, at time *t* is defined as follows:


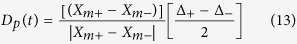



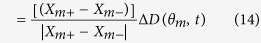



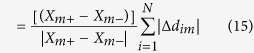


where a positive *D*_*p*_*(t)* implies a relaxing cell.

**Figure 5:**
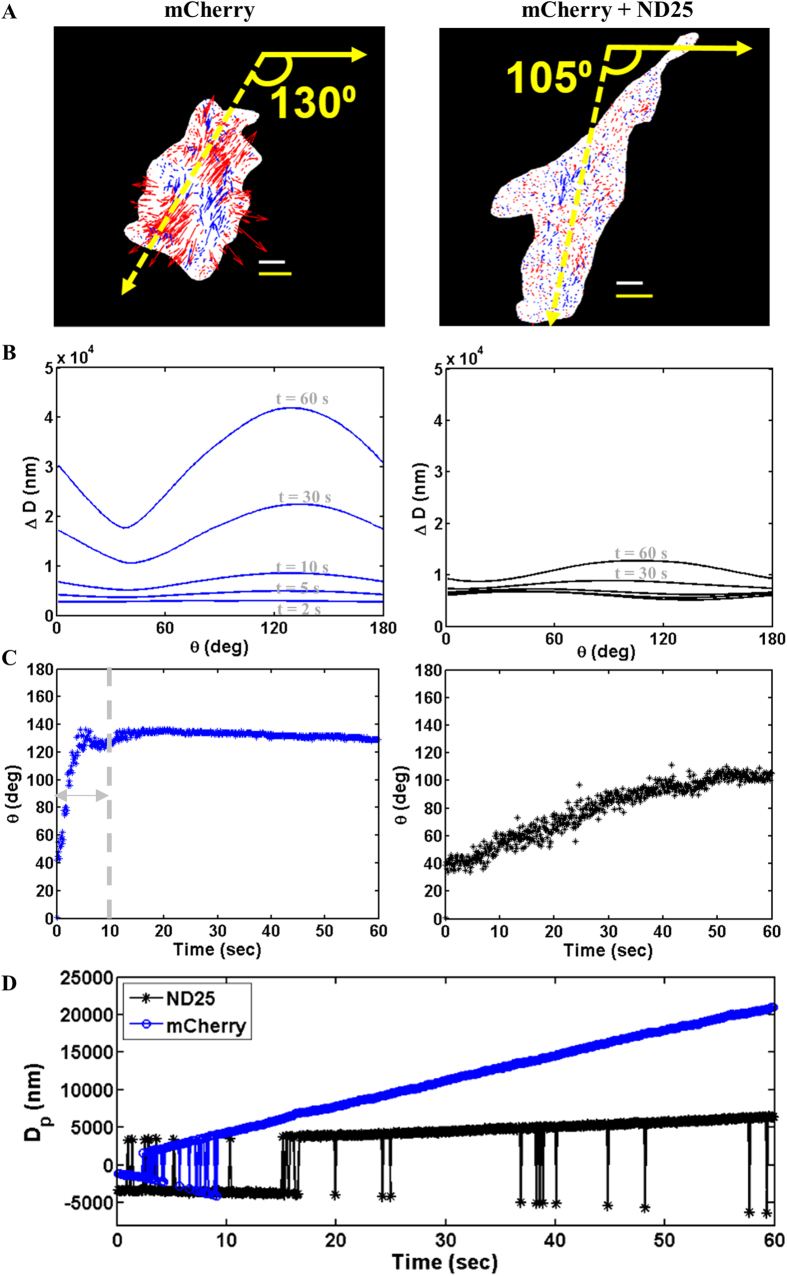
Time evolution of displacement dipole. Left and right column in (**A**–**C**) represent cells illuminated the mCherry and ND25 excitation light sources, respectively. (**A**) Displacements at *t* = 60 s show alignment (yellow) along *θ*, in accordance with dipole model indicated by *t* = 60 s on plot in (**B**). Arrows representing displacement magnitude are magnified 50x to aid visual clarity. Width of grey and yellow scale bars represent 10 μm and 0.25 μm, respectively. (**B**) Dipole strength at various time points (denoted in grey) during illumination. *t* = 2, 5, 10 s not denoted in right figure to preserve visual clarity due to overlapping curves. (**C**) Angle of dipole over 60 s-illumination period. Double headed arrow in left figure indicates time period during which dipole orientation is not well defined. Individual time points correspond to dipole strength curves in (**B**). (**D**) Displacement dipole value in terms of global displacement over time. Consistent positive D_p_ value after *t* ≈ 10 s for mCherry-illuminated cell indicates cell force relaxation after that point. (A negative D_p_ would indicate contraction). Fluctuating D_p_ for the ND25-illuminated cell indicates between force contraction and relaxation throughout the 60 s illumination period. The time periods in which the sign of D_p_ fluctuates (mCherry: 0–10 s, ND25: 0–60 s) represents the periods in which the dipole is not yet well defined, as indicated by the progression of peak development in (**B**). Figure shows results for *E* = 2 kPa gel.

The metrics developed here allow us to describe how cell force relaxation occurs over time under different illumination conditions. Geometrically polarized cells in which force distributions are expected to be highly aligned were utilized. This analysis technique highlights the difference between the effect of excitation by the fully intensity mCherry source and the mCherry + ND25 excitation light on cell forces (Fig. 5). Cells illuminated with the mCherry + ND25 show less relaxation (Fig. 5A). With the mCherry source, cells transition to a primarily relaxing state throughout exposure. The time-evolution of this photo-induced relaxation and displacement alignment is characterized by the evolution of the dipole orientation, as determined by the strength and alignment of the dipole. Dipole strength, Δ*D*_*m*_*(θ*_*m*_*, t)*, and orientation, *θ*_*m*_*(t),* were determined for all studied cells. Dipole strength represents the magnitude of cell-induced motion projected onto a unit vector at a given angle. Figure 5B shows distributions of Δ*D(θ, t)* at *t* = 2, 5, 30, and 60 s for a cell subjected to illumination continuously for 60 s (illumination protocol 1). The corresponding dipole orientation is determined by the maximum of Δ*D(θ, t)* (Fig. 5C). During the early phase of exposure (*t* = 0–10 s), the angle of maximum dipole strength is not well defined for cells (as shown by the overlapping curves for *t* = 2, 5, and 10 s in Fig. 5B). As illumination proceeds, the dipole orientation becomes well defined for cells illuminated by the full intensity mCherry source, and stabilizes at a constant value (Fig. 5B,C). The displacement dipole, *D*_*p*_*(t)*, exhibits continuous relaxation for cells illuminated by the full intensity mCherry source, which is consistent with the observation that displacements move outward (Fig. 5D). However, the cells exposed to mCherry + ND25 illumination exhibit much less relaxation over time. This large-scale relaxation was observed in all cells (n = 17) for each tested substrate stiffness. Surprisingly, the displacement dipole value at 60 s is similar for the cells exposed to light for only 2 s of the mCherry source (Fig. S6A,B). These results are consistent with the similarity between displacements observed after either 2 or 60 s of illumination (Fig. 2C). This suggests that the cells are affected by illumination within 2 s, and continue to reorganize applied forces at a rate comparable to those exposed continuously for one minute.

This analysis method illustrates that cells fluctuate between a globally contractile to relaxing state before achieving constant relaxation during illumination, and how that process differs temporally for different light sources. For the representative cell exposed to mCherry + ND25 excitation light in Fig. 5, the dipole strength is maximum at 105°. However, the distribution is broad and not sharply peaked (Fig. 5B). This suggests that the cell has not relaxed to the same extent that the cell illuminated with full intensity mCherry source has relaxed, when illuminated for the same amount of time as shown clearly in Fig. 5D. Additionally, the displacement dipole value for the mCherry + ND25 source continues to fluctuate between negative (contraction) and positive (relaxation) throughout illumination, while the same quantity fluctuates for only the first ten seconds of illumination after which it sustains a consistent positive value for the mCherry-exposed cell (Fig. 5D). This indicates that the cell exposed to the full intensity mCherry source achieves consistent force relaxation during of illumination. The ability to distinguish between dynamic cell contraction and relaxation during illumination is a quantitative technique for assessing photo-induced changes in cells prior to observable changes in morphology.

**Figure 6:**
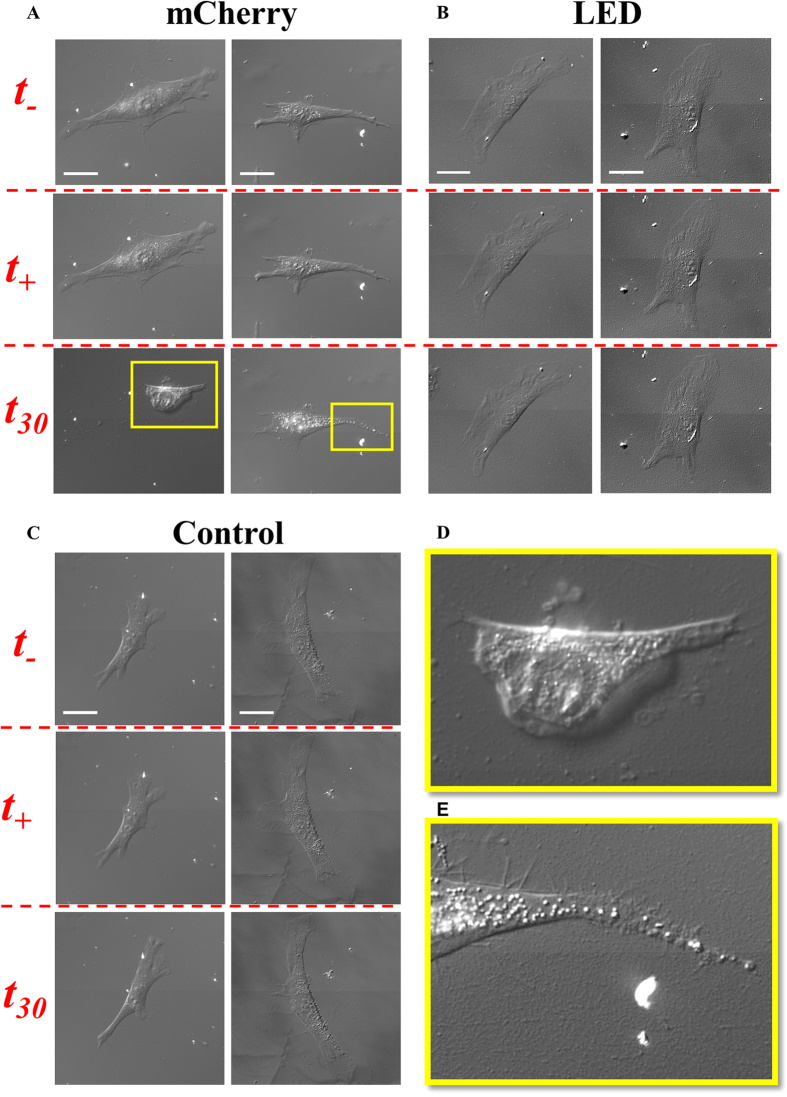
Change in morphology occurs long after fluorescent exposure and depends on excitation light. Morphology of two sample cells (n = 5) shown in DIC for each excitation light source ((**A**) widefield halide fluorescence + mCherry filter and (**B**) deep red collimated LED) and for (**C**) no fluorescence exposure (control), immediately before (t_–_, top row), after (t_+_, top row), and 30 min after (t_30_, bottom row) continuous illumination for 60 s. Significant morphology changes in (**A**) include decreased cell spread area (both cells) and spindle fiber formation and disorganization of nuclear region (right cell). Negligible morphology changes shown in (**B**). Enlarged view of morphology changes at t_30_ for left and right cell as indicated by yellow insets in (**A**) are shown in (**D**,**E**), respectively. All cells plated on PA gel of stiffness, *E*, = 2 kPa. Scale bar = 30 μm.

As cell morphology is commonly used to assess cell health during illumination, we explored photo-induced cell contractility changes revealed by the cell force dipole model. While we observe clear changes in cell contractility due to short duration fluorescence illumination, we do not observe any clear morphological changes. This demonstrates that morphology is not immediately affected by photoexposure, and is not a sensitive read-out of photo-induced cell change. However, morphological changes manifest over longer time periods following illumination. The severity of the changes depends on the excitation light. This response is consistent across all tested substrate stiffnesses. Cells (n = 50) subjected to 60 s of fluorescence illumination did not exhibit significant change in spread area of (+1.25% ± 3.2%) immediately after illumination. Several (n = 5) of these individual cells were monitored in DIC over a longer time period (30 min) following illumination for 60 s by both mCherry and LED excitation light sources (Fig. 6A,B). Control cells exposed only to DIC exhibited no visible change in morphology immediately before, after, or 30 minutes following illumination (Fig. 6C). All cells exposed to mCherry excitation light for 60 s exhibit significant morphological changes 30 minutes after illumination (Fig. 6A). Such changes include formation of spindle fibers, and/or membrane dissolution and vacuole formation (Fig. 6D,E). All cells exposed to the mCherry excitation light source exhibit clearly observable morphological changes and do not return to their initial spread state following illumination. In most cases, the cells significantly reduce their spread area such that they completely detach from the substrate and do not reestablish their spread morphology. This irreversible morphological response indicates that cells are no longer viable following 60 s exposure by the mCherry excitation light source. When cells are exposed to LED excitation light for 60 s, no morphological change is observed 30 minutes after illumination (Fig. 6B). This is consistent with the lower probability of force relaxation for cells exposed to the LED source (Table S1). Morphological changes do not appear immediately after illumination by either mCherry or LED excitation light source. Additionally, the long timescale of morphological change indicates that force relaxation precedes morphological reorganization following illumination. Thus, the dipole model is useful to describe the dynamics of photo-induced changes in cell behavior significantly before any morphological evidence of cell response to excitation light.

## Conclusion

Fluorescence microscopy is a commonly used tool for live cell imaging. However, the effects of exposing live cells to fluorescent excitation light have typically been assessed qualitatively by observation of cell morphology. We find that cells exhibit light sensitivity before observable morphological change by measuring the contractile dynamics of cells on elastic substrates. Our results show that cells relax their contractile force through outward displacement of the underlying substrate during fluorescent exposure. Cells illuminated for either 2 s or 60 s underwent similar relaxation dynamics, suggesting the process occurs shortly after exposure and proceeds irreversibly. Both force relaxation and morphology change depend on the excitation light. Morphological change appears much later than force relaxation, and hence is not a reliable real time indicator of photo-induced disruption to normal cell behavior. This work identifies cell force relaxation as a rapid effect of fluorescence illumination, and introduces a quantitative tool to characterize nanoscale relaxation dynamics before any observable changes in morphology.

## Additional Information

**How to cite this article**: Knoll, S. G. *et al.* Contractile dynamics change before morphological cues during florescence illumination. *Sci. Rep.* **5**, 18513; doi: 10.1038/srep18513 (2015).

## Supplementary Material

Supplementary Information
