# Corrigendum: Contractile dynamics change before morphological cues during fluorescence illumination

**DOI:** 10.1038/srep21218

**Published:** 2016-02-18

**Authors:** S. G. Knoll, W. W. Ahmed, T. A. Saif

Scientific Reports 5: Article number: 18513; 10.1038/srep18513 published online: 12222015; updated: 02182016.

The original version of this Article contained an error in the title of the paper, where the word “fluorescence” was incorrectly given as “florescence”. This has now been corrected in the PDF and HTML versions of the Article.

