# Supplementary material for: Contractile dynamics change before morphological cues during florescence illumination
Source: Sci Rep. 2015 Dec 22;5:18513. doi: 10.1038/srep18513 (PMC4686977; doi:10.1038/srep18513)
Supplement: Supplementary Information [file srep18513-s1.pdf]

Supplementary Information

Contractile dynamics change before morphological cues during fluorescence illumination

S. G. Knoll,[†] W. W. Ahmed,[‡] and T. A. Saif[†*]

†Department of Mechanical Science and Engineering, University of Illinois at Urbana-Champaign, Urbana, IL, USA
‡Laboratoire Physico-Chimie, Institut Curie, Centre de recherche, Paris, France; Sorbonne Universités, Université Pierre et Marie Curie, Paris, France; Centre National de la Recherche Scientifique, UMR168, Paris, France
*saif@illinois.edu

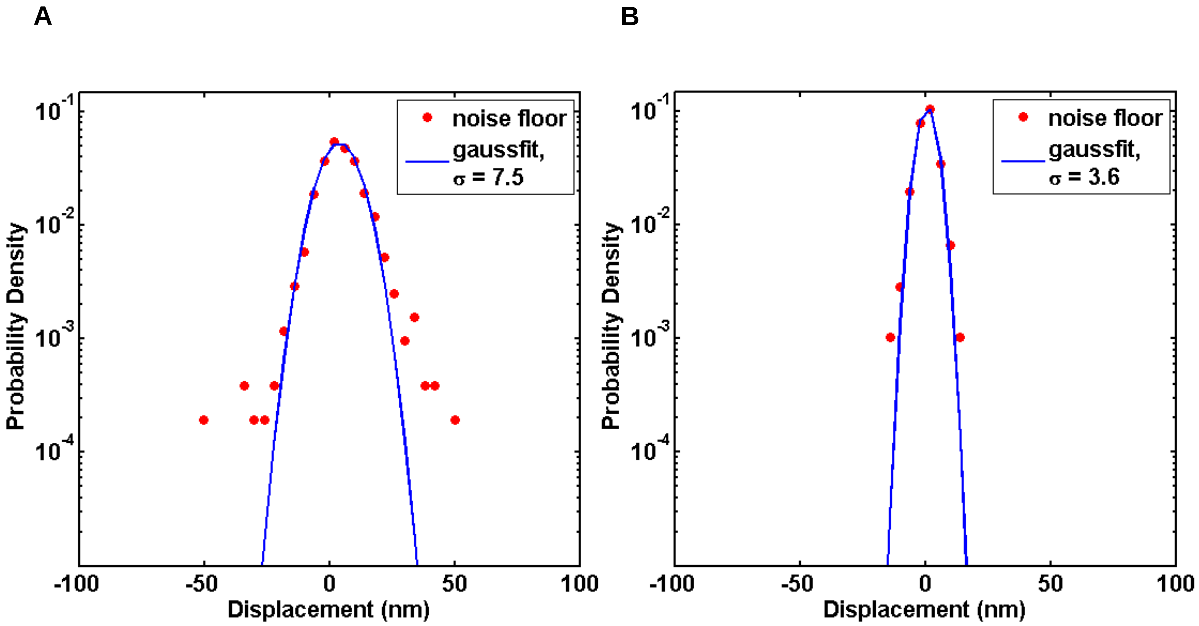

Fig. S1 Particle tracking precision for beads immobilized in PA gel substrates. Probability distributions for bead displacement in gels devoid of cells over one-minute illumination. Bead displacements on *(A)* 2 kPa and *(B)* 10 kPa substrates are shown in red. A Gaussian fit representing displacements of the noise floor is shown in blue. Displacements represent over 500 particles from 3 distinct gel substrates.

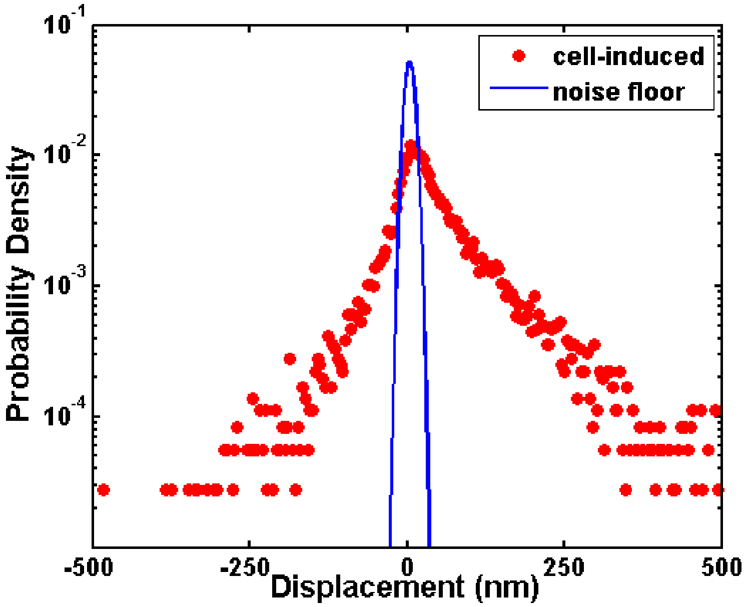

Fig. S2 Cell-induced displacements of stiff substrate represent cell force greater than thermal noise. Probability distributions for displacements over one-minute illumination period showing the magnitude of displacement of all points after 60 seconds. Displacements underlying cells (n=17) plated on 10 kPa substrate shown in red (σ = 15.7). Gaussian representing thermal displacements of beads in gel with no cells shown in blue (σ = 3.6).

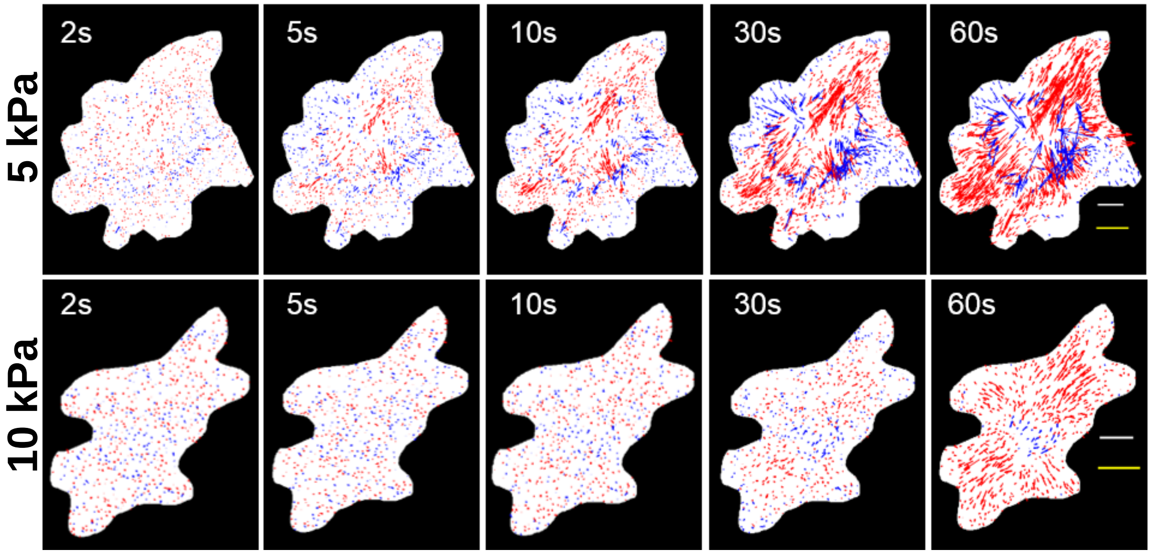

Fig. S3 Directionally preferential cell force relaxation on various stiffness substrates during illumination. Displacement changes as a result of changing cell forces during 60 s of continuous exposure. Blue and red arrows indicate inward and outward motion relative to the area centroid, respectively. Arrows representing displacement magnitude are magnified 50x to aid visual clarity. Gray and yellow scale bars pertain to scale of cell and arrows, respectively. Width of gray and yellow scale bars represent 10 μm and 0.25 μm, respectively.

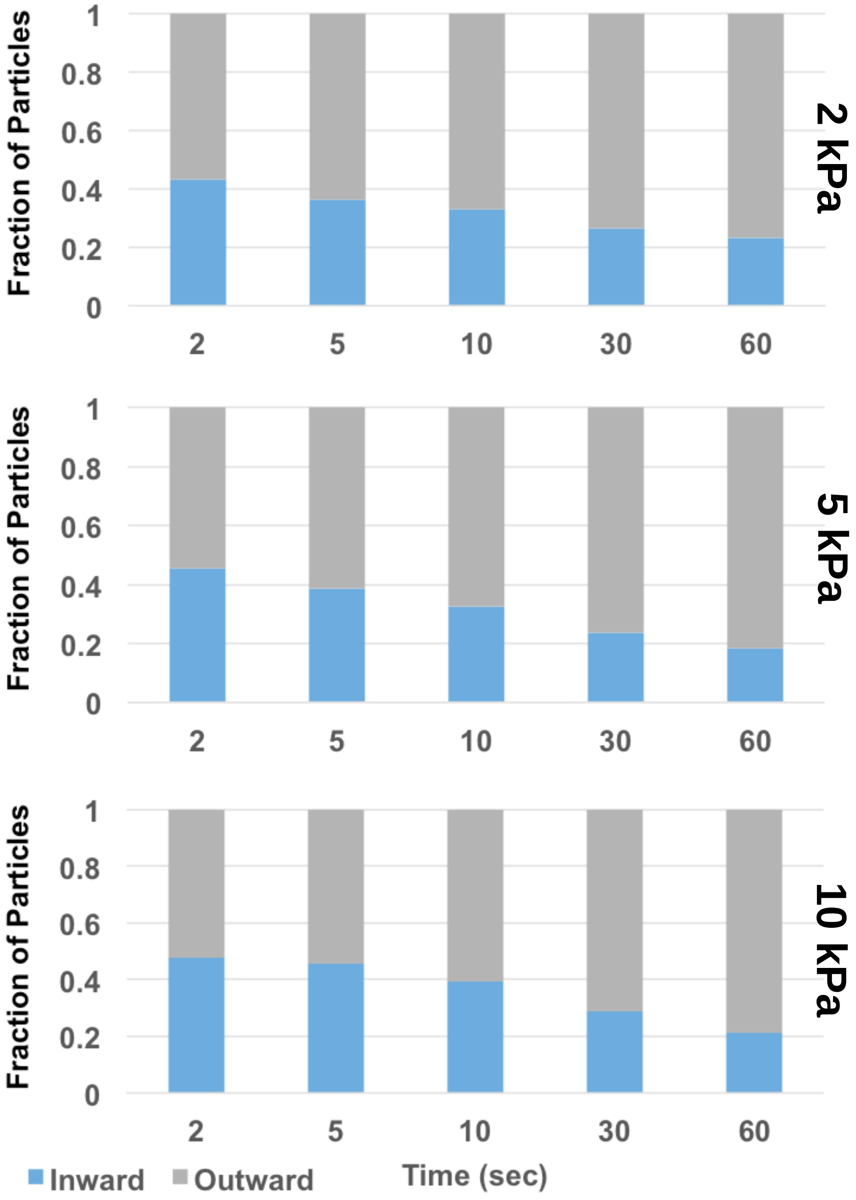

Fig. S4 Cell force relaxation increases with illumination time on various stiffness substrates during illumination. Proportion of outward- relative to inward- moving beads represents increasing dominance of force relaxation over contraction throughout illumination. Displacements induced by cells on 2 kPa (top), 5 kPa (middle), and 10 kPa (bottom) exhibit similar trends. Bar graph represents displacements of all beads for n=17 distinct cells.

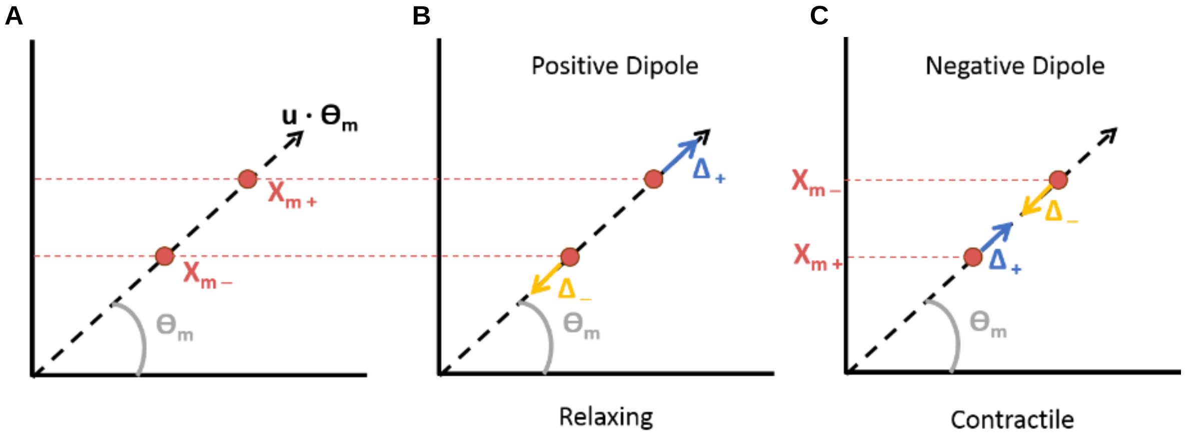

Fig. S5 Schematic of net displacement dipole. Net positive and negative displacements and their centroids are shown. Along the direction θ_m, the sum of the projected displacements (absolute values) maximize. *(A)* Bead displacements, Δx and Δy, were projected onto a unit vector along angle θ {1°, 180°}. Summed projected displacements at each time point were used to compute coordinates of the mean spatial locations, X_{m+} and X_{m-}, of the force dipole and the corresponding outward (Δ_+) or inward (Δ_-) displacements at those coordinates. The spatial location of X_{m+} relative to X_{m-} determines the forcing motif (*(B)*relaxing or *(C)* contractile).

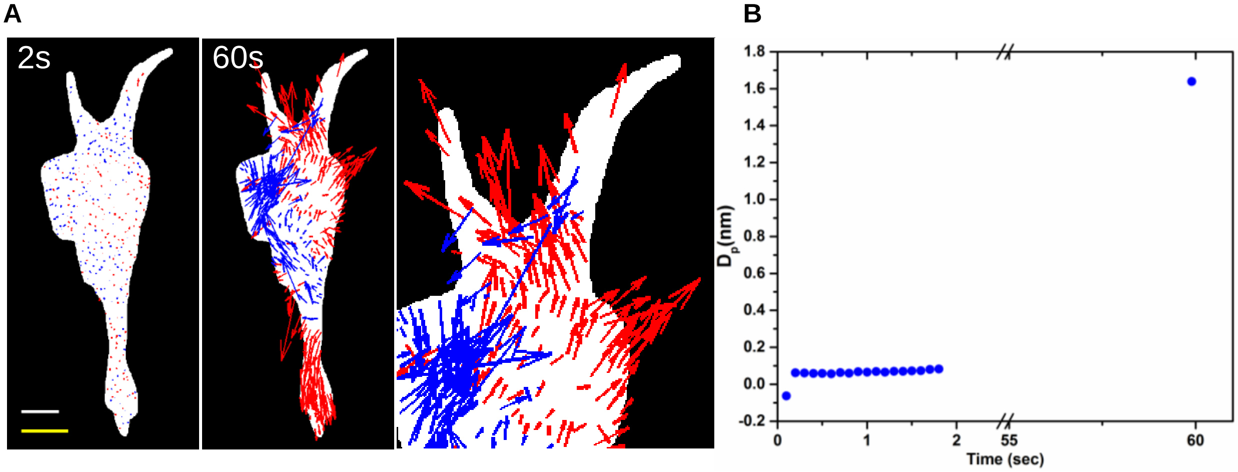

Fig. S6 Cell force reorganization initiates within 2s of illumination *(A)* Global displacements after 2 s (left) and at t=60 s (middle). Arrows representing displacement direction become aligned by the 60th second. Enlarged image (right) shows zoomed in region of displacements at t=60 s to emphasize alignment. Arrows representing displacement magnitude are magnified 50x to aid visual clarity. Gray and yellow scale bars pertain to scale of cell and arrows, respectively. Width of gray and yellow scale bars represent 10 µm and 0.25 µm, respectively. *(B)* Force dipole values for same cell in terms of global displacement over time. Here, dipole strength is measured for the first 2 s, and then at t=60 s, since the same light is used to fluoresce the cells and track bead displacements. Although the cell was only illuminated from t = 0 – 2 s, D_p increases between 2 and 60 s, which is consistent with trend shown in Fig. 6. All D_p represent values x 10^4. Break in x-axis represents t=2.5 – 55 s.

	Probability		
**Displacement Direction**	2 kPa	5 kPa	10 kPa
outward (relaxation)	0.75	0.82	0.74
inward (contraction)	0.25	0.18	0.26

Table S1 Displacement direction indicates cell force relaxation on various stiffness substrates. High (>0.5) probability of outward- and inward- moving cell-induced displacements relative to the cell centroid suggests cell force relaxation. Cell relaxation is observed on substrates of various stiffnesses (2, 5, and 10 kPa).

**Excitation Light**	***I* (W/m²)**	**λ (nm)**	**P(r)**
mCherry	12.5	540-585	0.77
mCherry + ND25	3.0	540-585	0.62
LED	1.9	640-680	0.53

Table S2 Specifications for various excitation light sources. Probability of outward- *(P(r))* moving bead displacements relative to the cell centroid increases with increasing illumination intensity, where the probability of inward motion, *P(c)*=1-*P(r)*.
